# Integrin β3 Is Required in Infection and Proliferation of Classical Swine Fever Virus

**DOI:** 10.1371/journal.pone.0110911

**Published:** 2014-10-23

**Authors:** Weiwei Li, Gang Wang, Wulong Liang, Kai Kang, Kangkang Guo, Yanming Zhang

**Affiliations:** 1 College of Veterinary Medicine, Northwest A&F University, Yangling, Shaanxi, China; 2 Jiangsu Key Laboratory of Biological Cancer Therapy, Xuzhou Medical College, Xuzhou, Jiangsu, China; Thomas Jefferson University, United States of America

## Abstract

Classical Swine Fever (CSF) is a highly infectious fatal pig disease, resulting in huge economic loss to the swine industry. Integrins are membrane-bound signal mediators, expressed on a variety of cell surfaces and are known as receptors or co-receptors for many viruses. However, the role of integrin β3 in CSFV infection is unknown. Here, through quantitive PCR, immunofluorescence (IFC) and immunocytohistochemistry (ICC), we revealed that ST (swine testicles epithelial) cells have a prominent advantage in CSFV proliferation as compared to EC (swine umbilical vein endothelial cell), IEC (swine intestinal epithelial cell) and PK (porcine kidney epithelial) cells. Meanwhile, ST cells had remarkably more integrin β3 expression as compared to EC, IEC and PK cells, which was positively correlated with CSFV infection and proliferation. Integrin β3 was up-regulated post CSFV infection in all the four cell lines, while the CSFV proliferation rate was decreased in integrin β3 function-blocked cells. ShRNA1755 dramatically decreased integrin β3, with a deficiency of 96% at the mRNA level and 80% at the protein level. CSFV proliferation was dramatically reduced in integrin β3 constantly-defected cells (ICDC), with the deficiencies of 92.6%, 99% and 81.7% at 24 h, 48 h and 72 h post CSFV infection, respectively. These results demonstrate that integrin β3 is required in CSFV infection and proliferation, which provide a new insight into the mechanism of CSFV infection.

## Introduction

Classical swine fever virus (CSFV) causing the disease of “classical swine fever (CSF)”, is a member of the Flaviviradea family which also contains Dengue fever virus, West Nile virus (WNV) and Hepatitis C virus. CSFV leads to huge economic loss in the swine industry as CSF causes problems characterized by high fever, alternating constipation, diarrhea and high mortality [1], [2]. Transmission of this virus mainly depends on contact spread, but recent investigations indicated that infected boars could transmit CSFV in semen as well [3], [4]. This disease was first identified in USA during a continuous major nationwide epizootics, and was subsequently found in many other countries [5], [6]. The last major CSF epidemic in European Union occurred mainly in Netherlands and to a lesser extent in Germany, Italy, Belgium and Spain [7], [8]. Considering its rapid speed in spreading across national borders, and the huge socio-economic damage to the porcine industry, CSF was classified as a notifiable disease by OIE [9].

CSFV entry into the host cell is through the receptor-mediated interaction of virus with cell membrane. Receptors for CSFV recognition are crucial for host antiviral immune responses [10]. Thus blocking CSFV receptors could be an effective way to control CSFV. Glycoprotein E (gpE), presented on the flaviviral particle surface has a conservative spatial structure [11]. It is thought to interact with cell surface receptors during the early stage of virus infection. Research done by Bogachek, demonstrated that the gpE domain III from WNV interacts with integrin αvβ3 at an initial stage of entry into the host cells [12]. However, specific function of integrin in CSFV infection has not yet been reported.

Integrins are heterodimeric trans-membrane adhesion molecules comprising a family of non-covalently linked 18 α and 8 β subunits [13]–[15]. It is also known that integrins play various roles in cell adhesion, migration, embryonic development, angiogenesis, tumor metastasis, immune responses and wound healing [16], [17]. Specifically, integrin β3, which is present on a variety of cell surfaces, is one of the most important cell receptors. Integrin β3 mediates extracellular signals by recognizing and delivering different molecules into cells, resulting in changes in calcium, Pyk2 (proline-rich tyrosine kinase 2) and PI-3K (Phosphoinositide 3-kinase) activity [18]–[26]. The activation and communication between PI-3K and internal calcium are crucial for integrin outside-in signaling. More importantly, integrin β3 is known to be a receptor or co-receptor for many viruses, such as Rotavirus [27]–[30], Hantavirus [31]–[33], and Foot-and-mouth disease virus [34], [35]. It also plays a key role in infection processes of Adenovirus [36], [37], West Nile Virus [32], Human immunodeficiency virus [38], [39], Human Parecho virus [40]–[42], and Herpes simplex virus type 1 [43]–[45]. Furthermore, previous studies from our laboratory have shown that integrin β3 is up-regulated in endothelial cells post CSFV infection (post CSFV infection = p.i.) [46].

Even though integrin is a demonstrated receptor or co-receptor for many viruses, little is known about the role of integrin in CSFV infection. Here, we demonstrate that integrin β3 is positively correlated with CSFV infection and proliferation, especially in ST cells. Functional-blocking or shRNA-mediated depletion of integrin β3 in EC cells dramatically decreased the rate of CSFV proliferation. This study also adds another receptor-function of integrin β3 and provides the first insight of a promising active receptor for CSFV infection. Furthermore, our results suggest that targeted inhibition or depletion of integrin β3 could be a potential therapeutic treatment for CSFV infection.

## Materials and Methods

### 1 Cell lines, reagents and CSFV infection

EC (swine umbilical vein endothelial cell) [47] and IEC (swine intestinal epithelial cell) [48] were immortalized porcine cell lines established and stored by our lab (Yanming, Zhang lab). PK (or PK-15, porcine kidney epithelial) [49] and ST [50] (swine testicles epithelial) cells were gifts from Harbin Veterinary Research Institute, Chinese Academy of Agricultural Sciences. SYBR Premix Ex Taq II (Perfect Real Time), Prime Script RT reagent kit with gDNA Eraser (Perfect Real Time) and One-step Prime Script RT-PCR kit (Perfect Real Time) were purchased from Takara (Dalian, China). Minimum Essential Medium (MEM) and G418 were products of Gibco (Carlsbad, CA, USA). Fetal calf serum was purchased from Hyclone (Logan, Utah, USA). Lipofectamine 2000 and Trizol reagent were from Invitrogen, Inc. (Carlsbad, CA). CSFV infected serum was a gift from Lanzhou Veterinary Research Institute. Mouse-anti-swine integrin β3 monoclonal antibody was purchased from Accurate chemical & scientific corporation (NY, USA). Horseradish peroxidase-conjugated goat anti-mouse IgG was purchased from Santa Cruz Bio. (USA).

Cells for all assays relating to CSFV infection were cultured to a confluent of 60% in 35 mm cell-culture dishes in MEM containing 10% fetal serum and then incubated with 1.5 ml CSFV liquid (100 CCID50/0.1 ml) at 37°C, 5% CO_2_ for 1 h. Subsequently, supernatant was discarded and the CSFV incubated cells were washed with fresh MEM to remove the unattached virions before the further cultivation in the final culture medium of MEM with 2% fetal serum at 37°C, 5% CO_2_.

### 2 One-step qRT-PCR and two-step qPCR

To establish the standard curve for one-step qRT-PCR, a 242-bp DNA was synthesized through PCR using standard primers of S-CSFV F and S-CSFV R (Table 1), designed based on CSFV coding sequence (GenBank accession number: AF091507.1). The standard CSFV RNA was obtained through reverse transcription from the synthesized 242-bp DNA, and then was quantified by Nano-Drop (ND-1000). The following formula: Copy number (copies/ml) = 6.02×10^23^ (copies/mol) × concentration (g/ml)/MW (g/mol) (MW = RNA length × 340 u) was used to calculate the amount of standard CSFV RNA. Thereafter the standard CSFV RNA was diluted in gradient liner ranging from 10° to 10^8^, and was used as normalized RNA samples to establish standard curve for one-step qPCR assay. One-step qRT-PCR was performed using One-step Prime Script RT-PCR kit (Perfect Real Time) in Thermal Cycler Dice Real Time System (Takara Bio., Dalian, China). The reaction mixture includes: 2 × one-step RT-PCR Buffer III, 12.5 µl; TaKaRa Ex Taq HS (5 U/µl), 0.5 µl; Prime Script RT Enzyme Mix II, 0.5 µl; PCR forward primer (10 µM), 0.5 µl; PCR reverse primer (10 µM), 0.5 µl; probe (10 µM), 0.5 µl; total RNA, 2 µg; RNase free H_2_O, added to 20 µl. One-step qRT-PCR assay was performed in a total volume of 25 µl using the recommended thermo cycling protocol with 42°C (reversely transcribe) for 20 min; 95°C (pre-denature) for 30 sec; followed by 35 cycles of 95°C (denature) for 15 sec, 57°C (anneal) for 20 sec, 72°C (extend) for 15 sec.

**Table 1:** Primers used in this paper.

Primers	Sequence (5′-3′)
S-CSFV F	ACTAATACGACGCACTATAGGGGTCTTTCACCAGGACTACAT
S-CSFV R	TTTTTTTTTTTTTTTTTTTTTTT TTCAGGGTTCTTGGCTCAC
CSFV F	GTTCTGCGAGGTGACCAAAAG
CSFV R	GATGCACACATAAGTATGGTAAAGC
CSFV Probe	(FAM) TCCGTCGCTACCTGTCACCCTACCT (Eclipse)
β3 qPCR F	CCATGATCGGAAGGAGTTTGCT
β3 qPCR R	AAGGTGGATGTGGCCTCTTTATAC
β-actin F	CGTCCACCGCAAATGCTTC
β-actin R	AACCGACTGCTGT CACCTTCAC

For two-step qPCR, primers of CSFV F and CSFV R (table 1) for CSFV quantification were designed based on CSFV coding sequence (GenBank accession number: AF091507.1) and primers of β-actin F and β-actin R (table 1) for house keeping gene of β-actin were designed based on the porcine β-actin coding sequence (GenBank accession number: DQ845171). Total RNA from different samples was harvested with Trizol reagent and was reversely transcribed to cDNA by using Prime Script RT reagent kit with gDNA Eraser (Perfect Real Time) (Takara Bio., Dalian, China). Reaction for reverse transcription was proceeded according to manufacturer’s instruction with the following procedures: 42°C (gDNA remove) for 2 min; 37°C (reversely transcribe) for 30 min; 85°C (inactive the reverse transcriptase) for 5 sec. Two-step qPCR was performed using SYBR Premix Ex Taq II (Perfect Real Time) in Thermal Cycler Dice Real Time System (Takara Bio., Dalian China) with the following procedures: 95°C (denature) for 3 min followed by 35 cycles of 95°C (denature) for 10 sec; 60°C (anneal) for 20 sec and 72°C (extend) for 20 sec. Fluorescence signal was auto-collected by Thermal Cycler at the end of each cycle. The individual sample was normalized for genome equivalents using the respective CT value of porcine β-actin. CT value stands for the minimum cycle number for the Thermal Cycler to reach the pre-set value of each tube’s fluorescence signal.

### 3 Evaluation of CSFV proliferation amount

For assessment of CSFV proliferation amount in cell-culture liquid, one-step qRT-PCR was used. Basically, samples of cell-culture liquid from four cell lines with CSFV challenge for different time periods (24 h, 48 h and 72 h) were harvested respectively. Subsequently, the amount of CSFV virions in cell-culture liquid from each group was assessed using one-step qRT-PCR as described in section 2 of materials and methods.

For evaluation of CSFV proliferation amount inside of the cells, two-step qPCR was used. Basically, total RNA from the four cell lines with CSFV challenge for 24 h, 48 h or 72 h were harvested and extracted as described before (section 2 of materials and methods). Procedures for the subsequent reverse transcription and two-step qPCR were the same with that mentioned in section 2 of materials and methods.

### 4 Immunofluorescence (IFC) and immunocytochemistry (ICC) assay

Cells challenged with CSFV for 24 h, 48 h and 72 h were fixed with 4% paraformaldehyde at room temperature (RT) for 20 min, followed by triple washing with phosphate-buffered saline (PBS). Then samples were treated with 0.1% triton X-100 for 10 min at RT and were subjected to another triple washing with PBS before blocking with 1% BSA for 1.5 h at RT. After the removal of BSA, another round of triple washing with PBS was followed. Subsequently, CSFV-infected swine serum was added as primary antibody to these prepared samples and incubated at 4°C overnight. After discarding the primary antibody, washing with PBS was performed three more times. Secondary antibody of horseradish peroxidase-conjugated goat anti-swine IgG (H+L) (Earthox, USA) for ICC and goat anti-swine IgG-FITC (5-isothiocyanato fluorescein) (Santa cruz Bio., USA) for IFC was then added to the primary-antibody labeled samples. After the incubation with secondary antibody at RT for 2 h, triple washing with PBS was followed. Subsequently, samples for IFC were observed under fluorescence microscope (Nikon Eclipse Ti), while samples for ICC were stained with AEC kit (BD Bio., USA) and then pictured under white light by Nikon Eclipse TE2000-U.

### 5 Quantitation of integrin β3 in CSFV-infected and CSFV-free cell lines

To evaluate the mRNA amount of integrin β3, two-step qPCR was performed using primers of β3 qPCR F and β3 qPCR R (Table 1) designed based on the swine integrin β3 coding sequence (GenBank accession number: AF282890.1). Briefly, total RNA from four cell lines with or without CSFV infection were extracted and reversely transcribed to cDNA as mentioned in section 2 of materials and methods. Reaction procedure for two-step qPCR was also the same as mentioned in section 2 of materials and methods.

### 6 Trypsin-digestion adhesion assay

To reveal the different adhesion abilities of the four cell lines, trypsin-digestion adhesion assay was performed. Basically, four cell lines were individually cultured to a density of 90% confluence in six-well plates one day before the digestion. Cells were then incubated with trypsin solution for 2 min at 37°C and observed using Nikon Eclipse Ti microscope. Trypsin solution was prepared by dissolving 0.25 g trypsin powder into 100 ml PBS.

### 7 Cell replication assay

Cell replication rates of four cell lines (EC, IEC, PK and ST) were evaluated by MTT assay. About 1×10^4^ cells were seeded into 96-well plates. After the cultivation for 12 h, 24 h, 48 h or 72 h, 20 µl MTT (5 mg/ml) was added to each well and incubated for an additional 2 h at 37°C. The culture medium was then replaced with DMSO (100 µl/well). Subsequently, absorbance value was evaluated at 570 nm by plate reader of Multiskan FC Microplate Photometer (Thermo scientific, USA).

### 8 Evaluation of CSFV proliferation rate in integrin β3 function-blocked cell

EC cells were incubated with mouse-anti-swine integrin β3 monoclonal antibody for 2 h to functionally block integrin β3 before CSFV challenge. In parallel, EC cells pre-incubated with non-specific IgG and infected with CSFV was set as the negative control group. After discarding the monoclonal antibody or non-specific IgG, cells were then washed with PBS and infected with CSFV following the protocol as mentioned in section 1 of materials and methods. RNA samples from cells incubated with monoclonal antibody or non-specific IgG for 24 h, 48 h or 72 h p.i. were then extracted and reversely transcribed to cDNAs. Two-step qPCR was used to evaluate CSFV proliferation in these antibody-pretreated cells (detailed procedure for reversely transcription and two-step qPCR were the same as that mentioned in section 2 of materials and methods.).

### 9 Establishment of integrin β3 constantly-defected cell (ICDC) and integrin β3 deficiency evaluation

To knockdown mRNA of integrin β3, four shRNA constructs (976, 1110, 1755 and nc, detailed sequence information can be found in Table 2) were synthesized by Gene Pharma (Shanghai, China). Nc construct generating none of any RNA hairpins was also designed as the negative control. All these constructs contain Neomycin and GFP genes, which are respectively responsible for G418-dependent cell selection and transfection-efficiency reporting.

**Table 2:** Parameters of the shRNAs.

Classification	Sequence
976 target	GCTGATAACTGAGAAGCTATC
976 shRNA	GCTGATAACTGAGAAGCTATCT*TCAAGAG*AGATAGCTTCTCAGTTATCAGCTTTTT
1110 target	GCAATGTCCTCCAGCTCATTG
1110 shRNA	GCAATGTCCTCCAGCTCATTGT*TCAAGAG*ACAATGAGCTGGAGGACATTGCTTTTT
1755 target	GGACTGGCTTCTACTGCAACT
1755 shRNA	GGACTGGCTTCTACTGCAACTT*TCAAGAG*AAGTTGCAGTAGAAGCCAGTCCTTTTT
nc target	GTTCTCCGAACGTGTCACGT
nc shRNA	GTTCTCCGAACGTGTCACGT*CAAGAGATT*ACGTGACACGTTCGGAGAATTTTT

To obtain ICDC, G418-dependent cell selection was performed. G418 is an aminoglycoside antibiotic used in cell selection to remove the non-transfected cell without Neomycin gene expression. In our EC-cell-based selection, 1 mg/ml of G418 worked out as an optimal condition resulting in more than 80% mortality of the non-transfected cells without affecting the transfected ones. Cells with shRNA or nc constructs transfected were cultured in the medium containing 1 mg/ml of G418 for at least 2 weeks. Then cells with fluorescence were passaged and seeded into 24-well plates. One week later, mono-colony cells with fluorescence formed small cell spots. At least 5 cell spots per well were picked up and seeded to a new 24-well plate. After another round of selection with G418 for at least 2 weeks, single spot of mono-colony cell was subjected to large-scale cultivation and was assessed according to the fluorescence density. Cells would subject to another round of selection until their fluorescence density reach 98% purity.

Two-step qPCR was used to determine the deficiency-efficiency on integrin β3 in ICDC or the cells with shRNA transiently transfected. Procedures for RNA extraction and reverse transcription as well as the system of two-step qPCR were the same as that mentioned in section 2 of materials and methods.

### 10 Evaluation of CSFV proliferation rate in integrin β3 function-blocked cell and ICDC

ICDC and integrin β3 function-blocked cells were infected by CSFV (refer to section 1 of materials and methods for detailed procedure of CSFV infection). Meanwhile, EC cells with non-specific IgG incubated or ICDC with nc construct constantly expressed were set as the negative control and infected by CSFV. RNA samples from these cells with CSFV challenge for 24 h, 48 h or 72 h were extracted and reversely transcribed to cDNA as mentioned in section 2 of materials and methods. Subsequently, two-step qPCR was used to evaluate CSFV proliferation rate in ICDC or integrin β3 function-blocked cells.

### 11 Western blot

Cells from four cell lines and ICDC were harvested and pelleted by centrifugation at 1,000 × g for 5 min at 4°C. The cells were washed once with PBS, and resuspended in lysis buffer that contained 2% sodium dodecyl sulfate (SDS) and denatured at 95°C for 5 min. The insoluble materials were removed by centrifugation at 13,000 × g for 20 min and the supernatant samples were loaded and separated by 12% SDS gel. After the blocking at room temperature for 1 h with 5% nonfat powdered milk in Tris-buffered PBS containing 0.05% Tween-20, the membrane was incubated overnight at 4°C with a 1∶500 dilution of the primary antibody of mouse-anti-swine integrin β3 monoclonal antibody (Accurate, USA) in a solution of PBS-T with 3% nonfat powdered milk. Then membranes were washed thrice in PBS-T for 10–15 min each time and incubated for 2 h at room temperature with a 1∶5,000 dilution of horseradish peroxidase-conjugated secondary antibody of goat-anti-mouse antibody (Santa Cruz, USA) in TBS-T with 3% nonfat milk. After three 15 min. washes with T-PBS, the samples were visualized using the Amersham ECL Prime Reagent (GE Healthcare, USA).

### Statistical analysis

Data from one-step qRT-PCR and two-step qPCR were collected in triplicate and assessed using 2^−ΔΔCt^ relative quantitative analysis. Statistical differences among means of two groups were calculated using student’s *t*-test. Differences between more than two groups were calculated using One-Way *ANOVA*. Western blot result was the mostly clear one from the three repeated performances, and quantified by using NIH ImageJ software.

## Results

### 1 CSFV proliferates best in ST cells

**Figure 1:**
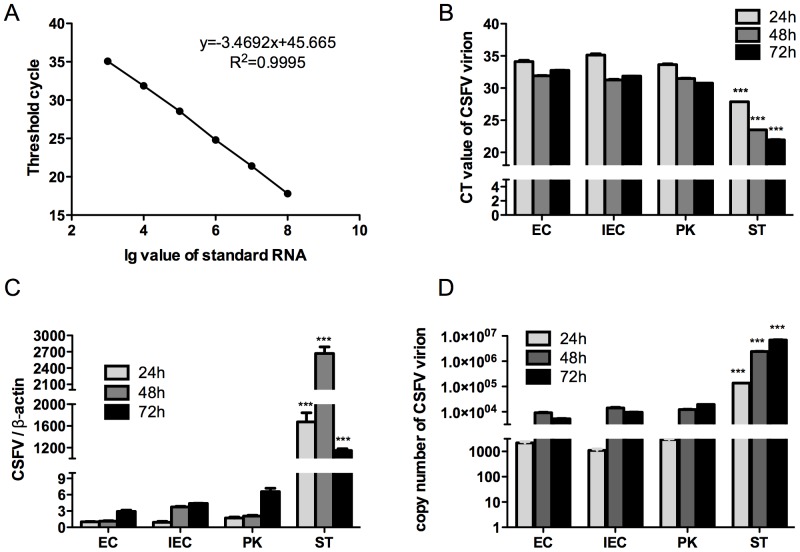
Establishment of standard curve for one-step qRT-PCR and evaluation of CSFV proliferation. **A)** Standard curve for one-step qRT-PCR. X-axis denotes Lg value of standard RNA and Y-axis denotes CT value (cycle for threshold, stands for the minimum cycle number of fluorescent quantitative PCR for Thermal Cycler to reach the defaulted value when collecting each tube’s fluorescence signal). The smaller the CT value is, the higher amount of interested RNA the sample contains. **B)** CT value from one-step qRT-PCR, showing the amount of CSFV virions in cell-culture liquid from four cell lines at 24 h, 48 h and 72 h p.i., respectively. **C)** CSFV proliferation amount in cell-culture liquid calculated according to the standard curve. The following formula is implemented: copy number = (45.665 - CT value)/3.4692. **D)** Evaluation of CSFV proliferation rate in four cell lines using two-step qPCR with β-actin as the norm. Statistical difference was calculated between ST group and EC, IEC or PK groups at the same time point, for example, *** marked above ST-24 column denotes extremely significant difference between ST-24 and EC-24, IEC-24 or PK-24 (This explanation applies to Fig. 1B, 1C and 1D).

As compared to the traditional two-step qPCR, one-step qRT-PCR minimized RNA degradation significantly since it integrates the steps of reverse transcription and quantitative PCR into a single reaction system. The standard curve for one-step qRT-PCR was well established and the parameters were E = 94.2%, R^2^ = 0.999, slope = −3.469 and y-int = 45.665, respectively (Fig. 1A). CSFV had the lowest CT value in cell-culture liquid from infected ST cells among all the four cell lines at every time point (Fig. 1B), indicating that ST cells have the highest ability in secreting CSFV virions to extracellular environments among all the tested cell lines.

The copy number of CSFV virions was calculated relative to the standard curve with the following formula: copy number = (45.665 - CT value)/3.4692. Fig. 1C shows the calculated amount of CSFV virions in cell-culture liquid from different cell lines at different time points (24 h, 48 h or 72 h) post CSFV challenge. CSFV proliferation amount in cell-culture liquid from ST cells was the highest and continually increased from 24 h to 72 h (Fig. 1C), indicating that CSFV virions secreted from ST cells were much more than that from the other three cell lines. In addition, the amount of CSFV virions secreted to cell-culture liquid had a generally increasing trend from 24 h to 72 h in all the four cell lines. As shown in Fig. 1D, the amount of CSFV proliferated in ST cells was much more than that in the other three cell lines. Specifically, the feature of CSFV proliferation was different in different cell lines from 24 h to 72 h p.i. In ST cells, CSFV reached its peak value at 48 h p.i., while it took 72 h for the other three cell lines to reach their peak values. This result suggests that EC, IEC, PK cells have a lower ability in secreting CSFV virions to extracellular matrix than ST cells do (Fig. 1C and 1D).

In summary, Fig. 1B, C, D reveal that CSFV proliferates better in ST cells than in the other tested cell lines. CSFV also has the highest amount in cell culture liquid from infected ST cells (Fig. 1B and 1C), implying that CSFV could be secreted into the extracellular environment more easily by ST cells than by the other three cell lines. This high affinity of CSFV in binding to ST cells may due to the high amount of integrin β3 expressed on ST cell membrane.

### 2 CSFV has the highest proliferation amount in ST cells confirmed by IFC and ICC

**Figure 2:**
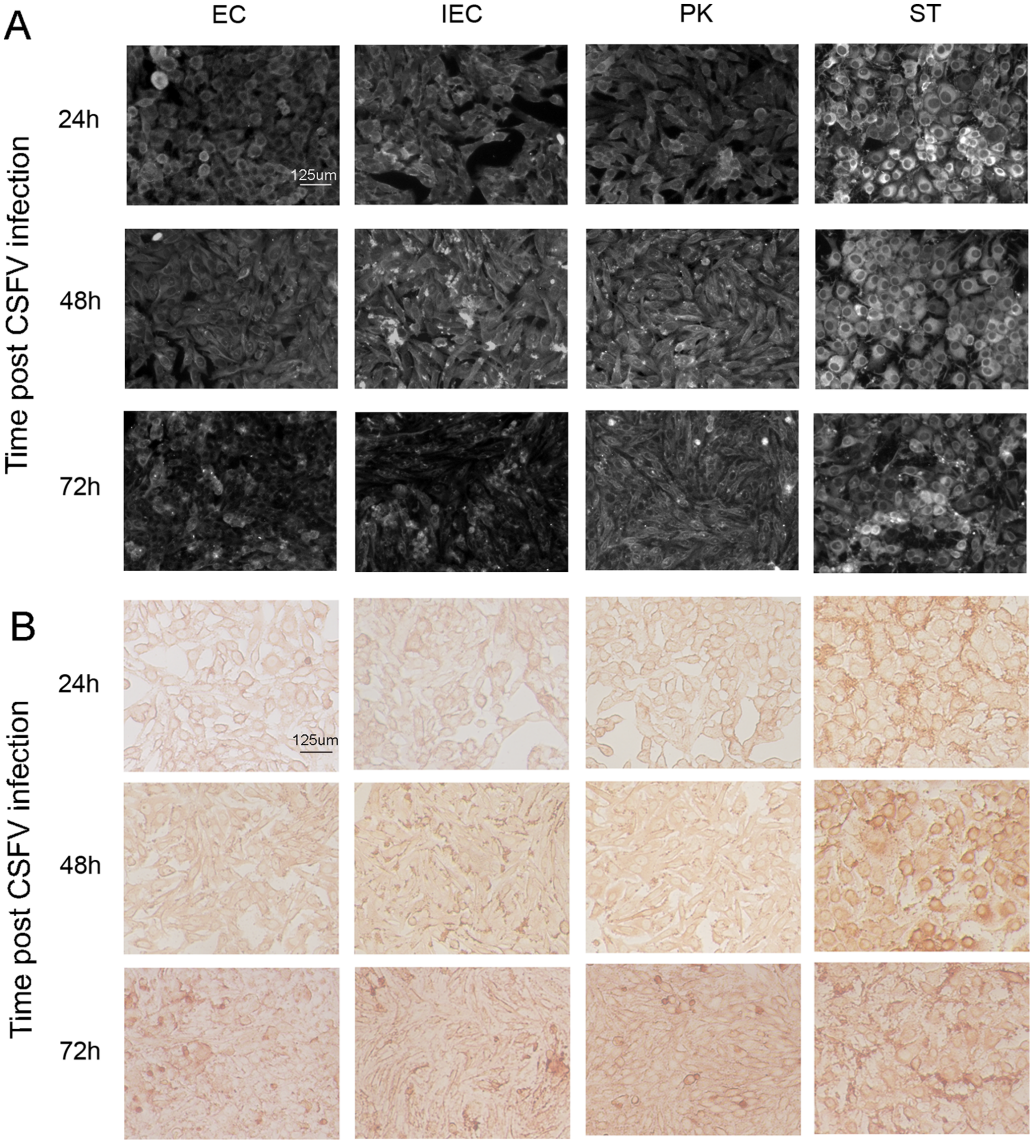
Immunofluorescence (IFC) and immunocytochemistry (ICC) results for evaluation of CSFV proliferation amount. **A)** IFC result of CSFV proliferation amount in the four cell lines p.i. The horizontal panels are different cell lines with CSFV challenge while the vertical ones represent different time points post CSFV challenge. The white spots denote CSFV virions, the brighter the spot is the more CSFV virions the cell contains. **B)** ICC result of CSFV proliferation amount in different cell lines p.i. The same with Fig. 2A, the horizontal and vertical panels denote different cell lines and different time points post CSFV challenge, respectively. The brown spots denote CSFV virions. The deeper the color is, the more amount of CSFV virions the cell contains. The exposure time for IFC and the background for ICC were kept consistent within each set of expreiments.

To obtain visualized evidence of ST cell’s predominant advantage in CSFV infection and proliferation, IFC and ICC assays were performed to assess CSFV virion amount in all four cell lines. As seen in Fig. 2A and 2B, CSFV had obviously higher proliferation amount in ST cells than in the other cell lines at each time point (24 h, 48 h or 72 h), consistent with the qRT-PCR results shown in Fig. 1C and Fig. 1D.

### 3 Integrin β3 has the highest amount in ST cells and is up-regulated after CSFV infection

**Figure 3:**
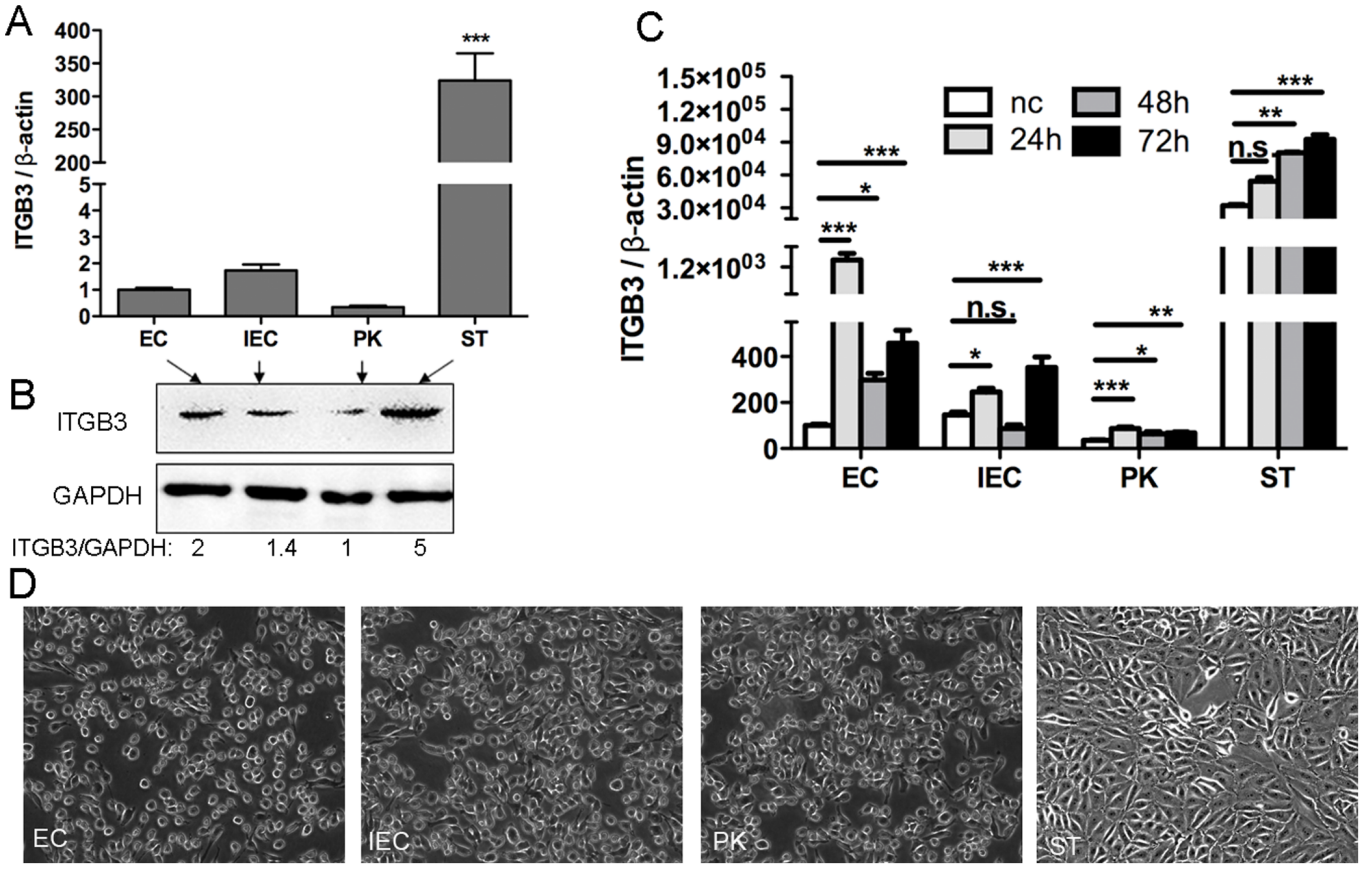
Assessment of integrin β3 amount and result of trypsin-digestion adhesion assay. **A)** Relative mRNA amount of integrin β3 in different cell lines before CSFV infection evaluated through two-step qPCR. ITGB3 denotes integrin β3. *** marked above ST column denotes extremely significant difference between ST and EC, IEC or PK. **B)** Western blot analysis of the amount of integrin β3 expressed in four cell lines. GAPDH denoting the house keeping gene of Glyceraldehyde 3-phosphate dehydrogenase, were used as the loading control. Ratio of the signal intensity was calculated through NIH ImageJ. **C)** Relative mRNA amount of integrin β3 in different cell lines after CSFV infection, compared to nc group. “nc” denotes the cell without CSFV infection. (***p<0.001, extremely significant difference; **p<0.01, very significant difference; *p<0.05 significant difference, n.s., no significant difference). **D)** Results of trypsin-digestion assay for cell adhesion. Cells were pictured after the incubation with 0.25% trypsin solution at 37 °C for 2 minutes. Cells with round edge indicated that they were digesting off from the ECM.

The amount of integrin β3 mRNA in all four cell lines (EC, IEC, PK and ST) was evaluated using two-step qPCR. As indicated in Fig. 3A, the amount of integrin β3 mRNA in ST cells was notably higher than that in the other three cell lines. Western blot analysis of integrin β3 protein levels (Fig. 3B) also demonstrated that ST cells had the highest amount of integrin β3 protein, in line with the result of two-step qPCR. Seen in Fig. 3B, ST cells have 5 times of the amount of expressed integrin β3 than that observed in PK cells. Moreover, integrin β3 mRNA was up regulated in all four cell lines after CSFV infection (Fig. 3C), revealing that integrin β3 is up-regulated by the host cell as a response to CSFV infection. Specifically, the result of Fig. 3C showed that ST cells and IEC cells exhibited the highest amounts of integrin β3 mRNA at 72 h p.i., while EC cells and PK cells reached peak amounts of integrin β3 mRNA at 24 h p.i. These results reveal that, as a response to CSFV invasion, integrin β3 is modulated to different levels in different cell lines. This may due to different cell lines having different sensitivity to CSFV infection, leading to various responses when proliferated CSFV virions accumulate inside cells.

It is well known that integrin β3 plays an important role in cell adhesion to the extracellular matrix (ECM). To determine whether different amounts of integrin β3 could reflect the correlative capacity in cell adhesion *in vitro*, we performed a trypsin-digestion adhesion assay. The results in Fig. 3D showed that EC, IEC and PK cells were digested off from the ECM and turned round in shape after incubation with trypsin solution for 2 minutes at 37°C. In contrast, ST cells changed little after the same digestion period. This result reveals that ST cells possess much higher adhesion ability to ECM than do the other three cell lines This is also in accordance with the results shown in Fig. 3A and 3B that ST cells have the highest amount of integrin β3. These results (Fig. 3A, 3B and 3D) are consistent with the accepted role of integrin β3 in cell adhesion.

To sum up, our results from two-step qPCR, Western blot and trypsin-digestion adhesion assays clearly confirm that ST cells have the highest amount of integrin β3 among all the tested four cell lines. These results also demonstrate that integrin β3 has different expression levels in different cell lines and can be up-regulated post CSFV infection.

### 4 CSFV proliferation rate is not related to the replication capacity of the host cell

**Figure 4:**
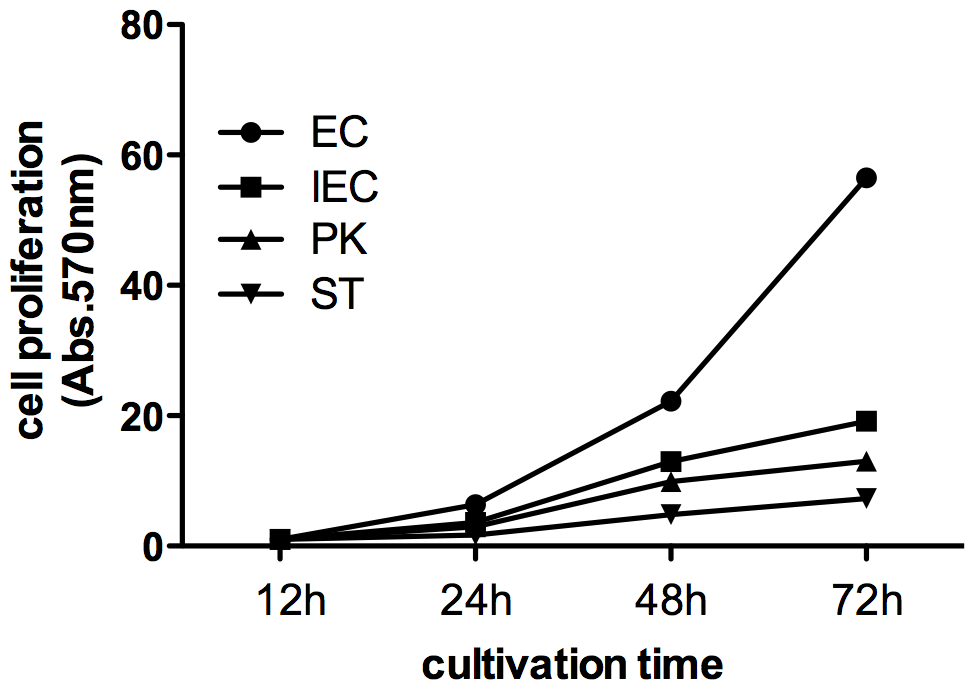
Cell replication assay. Cells were cultivated for 12 h, 24 h, 48 h and 72 h in 96-well plate before adding 100 µl MTT (5 mg/ml) per well. Absorbance value for each well was collected at 570 nm using Multiskan FC Microplate Photometer. Y-axis denotes relative absorbance value at 570 nm while the absorbance value at 12 h was set as 1.

The cell replication capacities of the four cell lines were assessed using the MTT assay in order to determine whether CSFV proliferation was affected by the growth rates of the host cells. As shown in Fig. 4, ST, IEC and PK cells replicated at similar rates, reaching peak MTT values at 48 h. By contrast, EC cells increased in number from 12 h to 72 h with peak amounts observed at 72 h. Interestingly, ST cells that have a predominant advantage in CSFV proliferation also had the lowest cell replication rate (Fig. 1 and Fig. 2). This result indicates that CSFV proliferation does not correlate with the replication capacity of the host cell.

### 5 CSFV proliferation rate decreases in integrin β3 function-blocked cell

**Figure 5:**
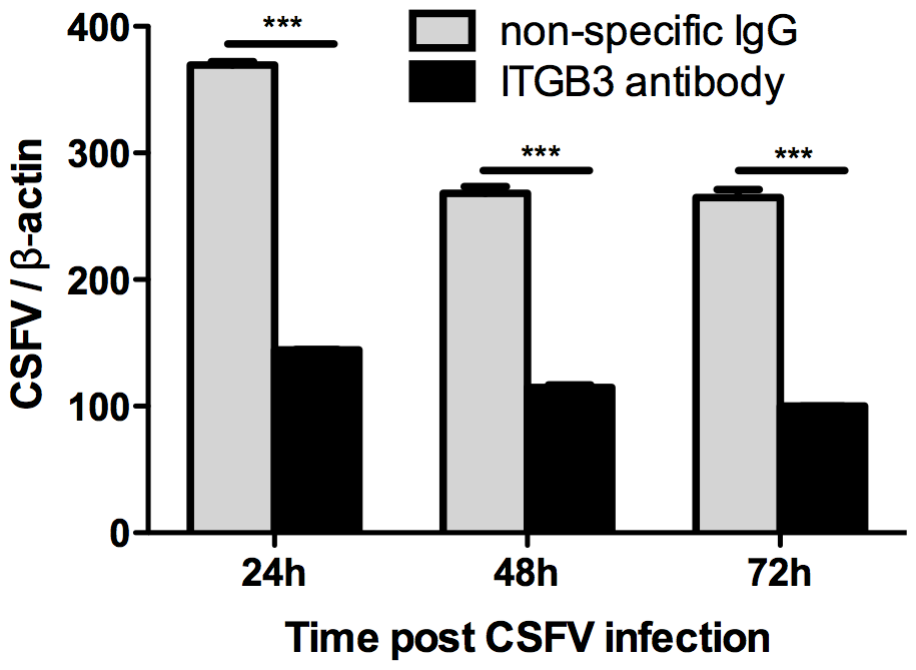
Evaluation of the CSFV proliferation rate in integrinβ3 functionally blocked cells. Y-axis denotes the normalized amount of proliferated CSFV assessed by two-step qPCR while X-axis denotes different time points p.i. Cells were pre-incubated with mouse-anti-swine integrin β3 monoclonal antibody at 37°C for 2 h before CSFV infection. Cells incubated with the non-specific IgG were used as the negative control. ***p<0.001 represents extremely significant difference.

Integrin β3 can be functionally blocked by mouse-anti-swine integrin β3 monoclonal antibody, allowing the evaluation of CSFV proliferation in cells with functionally blocked of integrin β3. The results in Fig. 5 demonstrate that CSFV has a lower proliferation rate in cells that were pre-incubated with mouse-anti-swine integrin β3 monoclonal antibody than in cells that were pre-incubated with non-specific IgG. The amount of CSFV proliferation in cells blocked by mouse-anti-swine integrin β3 antibody decreased to half of that observed in non-specific IgG blocked cells with the decline most obvious at 24 h p.i. Functional blocking of integrin β3 reduces CSFV proliferation (Fig. 5), implying that integrin β3 can affect CSFV infection and proliferation, presumably by affecting CSFV’s binding to the host cell membrane. In summary, our results (Fig. 1–5) indicate that the amount of integrin β3 expression is positively correlated to CSFV proliferation, demonstrating that integrin β3 affects CSFV infection and proliferation. This presumably occurs by integrin β3 involvement in CSFV internalizing to or secreting from the host cell.

### 6 Cells with shRNA1110 and shRNA1755 transiently transfected show moderate deficiency of integrin β3

**Figure 6:**
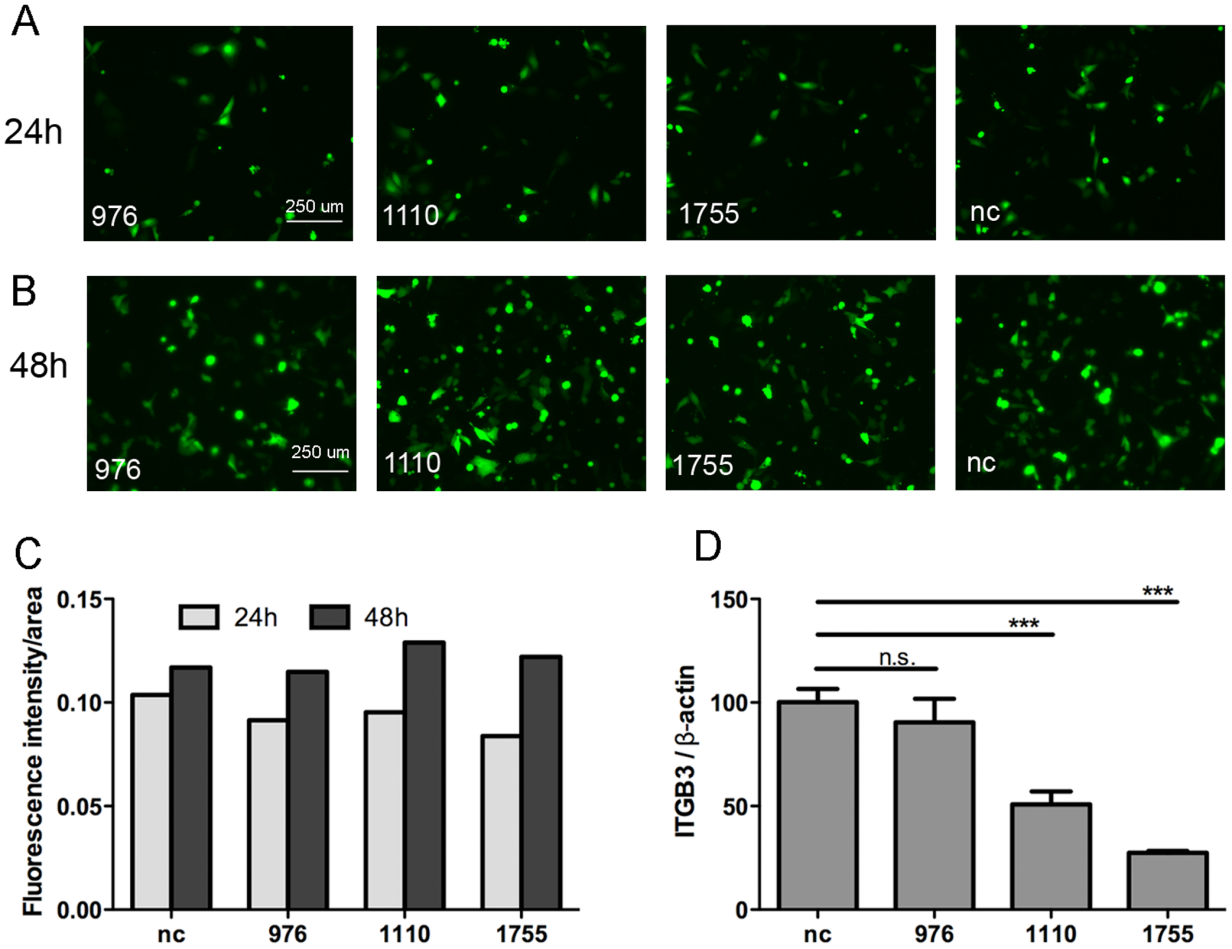
Evaluation of shRNA transfection-efficiency and integrin β3 deficiency. **A)** Fluorescence pictures of cells with shRNAs transiently transfected for 24 h. 976, 1110 and 1755 denote the targeting positions on integrin β3 mRNA sequence. nc denotes the negative-control construct which generates non-specific interfere RNA. **B)** Fluorescence pictures of cells transiently transfected with shRNA976, shRNA1110, shRNA1755 or nc for 48 h. **C)** Quantitative analysis of fluorescence intensity in Fig. 6A and 6B evaluated by ImageJ. **D)** Assessment of integrin β3 deficiency at 48 h post shRNA transiently transfection using two-step qPCR. ***p<0.001 denotes extremely significant difference. n.s. denotes no significant difference.

ShRNA silences target gene expression by generating short RNA pieces and forming a hairpin loop with the host cell mRNA. Here, shRNA976, shRNA1110, shRNA1755 were designed to interfere integrin β3 mRNA on specific gene positions. As a control, the nc plasmid generates non-specific small RNAs targeting none of the swine integrin β3 genes. In addition, GFP gene responsible for transfection-efficiency reporting and neomycin gene for G418-dependent cell-selection were added to each construct. The GFP analysis in Fig. 6A and 6B, revealed that shRNAs were successfully transfected and expressed in swine cells as observed at 24 h and 48 h post transfection. Cells transfected with different shRNAs had similar fluorescence intensity (Fig. 6C), revealing that the transfection efficiencies of different shRNA constructs were comparable. Cells had higher GFP expression level at 48 h than that at 24 h post shRNA transfection (Fig. 6C), indicating that 48 h post shRNA transfection is the optimal time to test the effect of the deficiency of integrin β3. Integrin β3 had deficiencies of 50% and 70% in cells transiently transfected with shRNA1110 and shRNA1755, respectively, as compared to the nc group (Fig. 6D). These results (Fig. 6A, B, C and D) indicated that all of the shRNA constructs, as well as the nc one, had comparable transfection efficiencies. Furthermore, shRNA1110 and shRNA1755 could decrease integrin β3 mRNA to a moderate level at 48 h post shRNA transfection.

### 7 CSFV proliferation rate decreases in ICDC

**Figure 7:**
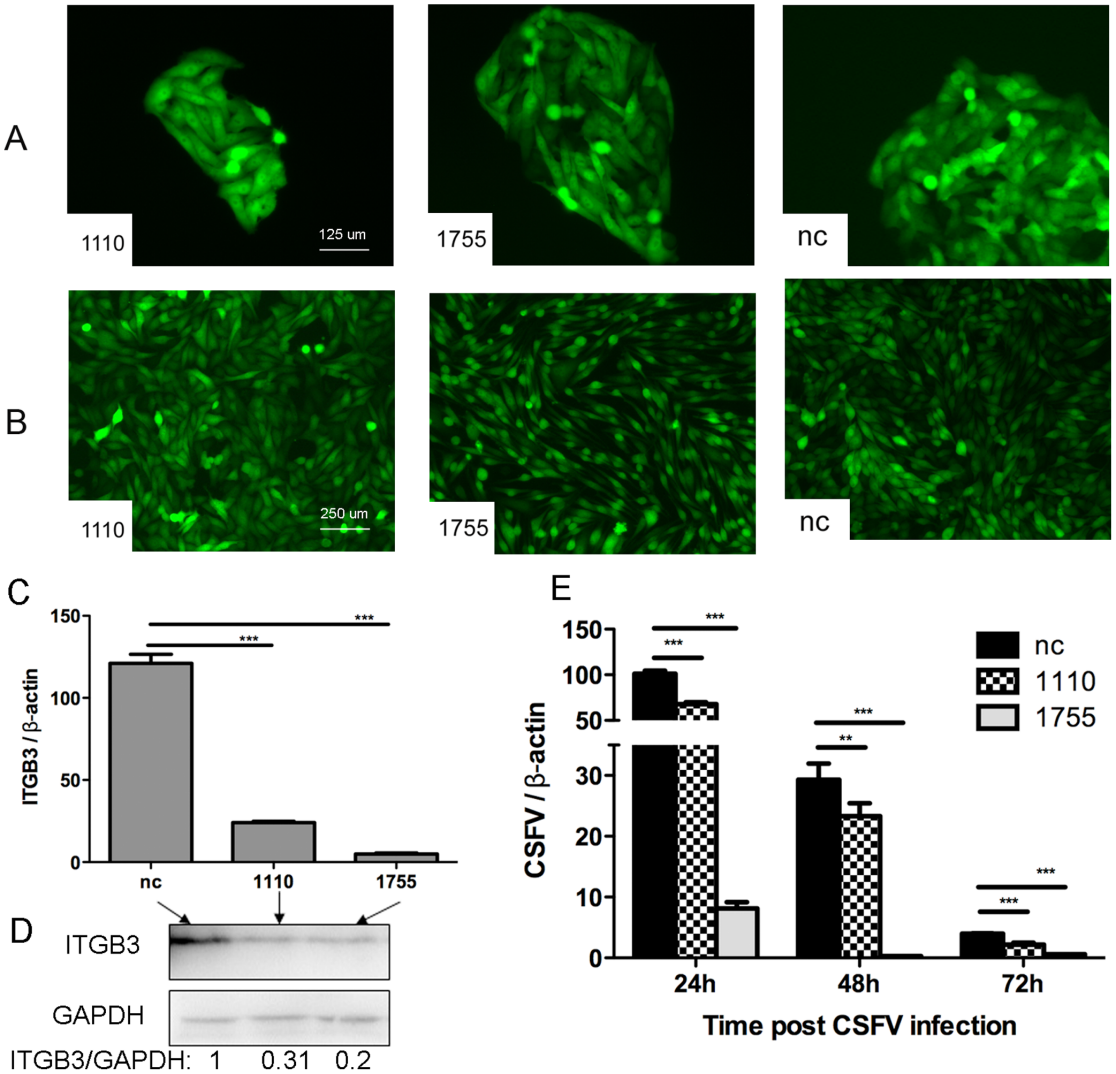
Evaluation of integrin β3 deficiency and CSFV proliferation in integrin β3 constantly defected cells (ICDC). **A)** Fluorescence pictures of ICDC colonies. 1110, 1755 and nc respectively denote the ICDC with shRNA1110, shRNA1755 or nc construct constantly expressed. **B)** Fluorescence pictures of mass cultures of ICDC used for CSFV infection. **C)** Assessment of integrin β3 deficiency in ICDC by two-step qPCR using β-actin as the norm. **D)** Western blot results showing the integrin β3 deficiency in protein level. Ratio of the signal intensity was calculated through NIH ImageJ. **E)** Evaluation of CSFV proliferation rate in ICDC at 24 h, 48 h or 72 h p.i., respectively. “nc” denotes CSFV proliferation rate in ICDC constantly expressing nc construct. (***p<0.001 denotes extremely significant difference, **p<0.01 denotes very significant difference).

Mono-colony cells constantly expressing shRNA1110 and shRNA1755 with a purity of more than 98% were successfully screened and cultivated on a large scale as shown in Fig. 7A and 7B. Considering that shRNA976 had little effect in interfering integrin β3 (Fig. 6D), we only selected the ICDC expressing shRNA1110 or shRNA1755 using G418. Correspondingly, deficiency of integrin β3 in ICDC was evaluated using two-step qPCR. ICDC expressing shRNA1110 and shRNA1755 significantly decreased the mRNA amount of integrin β3 with the deficiencies of 80% and 96%, respectively (Fig. 7C), as compared to the nc group (nc stands for the ICDC with nc construct constantly expressed). As shown in the Western blot results (Fig. 7D), the expression of integrin β3 decreased to 31% and 20% of that of the negative control group. Fig. 7C and 7D reveal that the efficiency of shRNA in reduction of integrin β3 is significant at both the mRNA and integrin protein levels.

The CSFV proliferation rate in ICDC was evaluated using two-step qPCR. ShRNA1755 constantly-expressed cells decreased CSFV proliferation significantly with deficiencies of 92.6%, 99% and 81.7% at 24 h, 48 h and 72 h p.i. (Fig. 7E), respectively, as compared to the nc group (nc stands for the ICDC without CSFV infection). This result is consistent with the integrin β3 functional blocking assay, thereby confirming that a cellular reduction of integrin β3 amount decreases the rate of CSFV proliferation. Collectively, these results imply that integrin β3 affects CSFV infection and proliferation *in vitro*.

## Discussion

Previous researches have demonstrated that integrin β3 is a cell-surface binding site for many viruses [27], [51]–[58], but little is known about the role of integrin β3 during CSFV infection and proliferation. In this study, we document that integrin β3, a membrane-bound signal mediator, is required for CSFV infection and proliferation. CSFV proliferates very efficiently in ST cells (Fig. 1 and 2), which was positively correlated with the high amount of integrin β3 in these cells (Fig. 3). In addition, the amount of CSFV proliferation decreased in integrin β3-funtionally blocked cells (Fig. 5) as well as in integrin β3-deficient cells (Fig. 7). Collectively, our results strongly imply that integrin β3 plays a critical role in CSFV infection and proliferation, presumably by modulating CSFV adherence and emergence from host cells.

Our observations that integrin β3 has a role in CSFV infection and proliferation also adds another set of virus-integrin-β3 interactions that are relevant to the receptor-function of integrin. Many reports revealed that viruses from Flaviviradea family use integrin β3 as a receptor or co-receptor during the infection stage [27], [51]–[58]. For example, acute CSF shares a high level of similarity with Dengue virus (DV) [2], [59] that causes classical dengue fever (DF) and dengue haemorrhagic fever/dengue shock syndrome (DHF/DSS) [53]. It has been demonstrated that integrin β3 is required for DV type2 to enter vascular endothelial cells, and that integrin β3 is up-regulated during DV infection [59], [60]. Similarly, we found that integrin β3 was up-regulated in all four swine cell lines (EC, IEC, PK and ST) during CSFV infection (Fig. 3C), which is in line with previous research of Tang, etc. [46]. ST cells possessing high amount of integrin β3, also show high susceptibility to CSFV infection. Many other studies have also reported that integrin β3 is a receptor for foot-and-mouth disease virus [54]–[56], coxackievirus A9 [51], echovirus 9 [52] and rotavirus [27], [30], [61]. In addition, adenovirus-mediated gene delivery to intestinal epithelium relies on, or is enhanced by the presence of integrin αvβ3 [62], [63].

Integrin β3 non-covalently binds with integrin αv subunit to form a heterodimeric complex. Integrin αv is also reported to play a role in virus infection, but the receptor-function of integrin αv is not as effective as integrin β3. Song, etc. [64] demonstrated that integrin β3 is important for Hantavirus infection, whereas integrin αv showed less effect than integrin β3 does *in vivo*. Neff, etc. [65] demonstrated that the bovine integrin β3 subunit was responsible for the receptor-function of integrin in FMDV infection, while αv subunit was not equally required as β3 subunit. Based on these prior reports, we focused on the role of integrin β3 in CSFV infection. Even though it is yet to be established why CSFV targets integrin β3, Bensaude, etc. have demonstrated that CSFV inhibits apoptosis of host cells, thus allowing the establishment of long-term infection and proliferation [66]. Integrin is responsible for cell adhesion, so its depletion would lead to loss of adhesion and subsequent apoptosis. The results from our study and Tang, etc. confirm that integrin β3 is up-regulated post CSFV infection. This suggests that CSFV infection results in the up-regulation of integrin so as to keep host cells in a condition that allows additional proliferation of virions.

CSFV contains 4 structural proteins: C, Erns, E1 and E2. Erns and E2 is the glycoprotein expressed on the surface of virus envelope, inducing the immunity response of the host animal. Heparin sulfate (HS) was previously reported to be the receptor for Erns [67], [68], but it was also demonstrated that there are other strains of CSFV not dependent on HS to infect host cells. This indicates that the receptor for E2, which has not yet been disclosed, is much more specific and important for CSFV infection. Bogachek [12] demonstrated that gpE from WNV recognizes integrin αvβ3 during the entry stage into the host cells. In this study, we demonstrated that integrin β3 is indeed required for CSFV infection and proliferation. This indicates that integrin β3 could be a promising receptor for glycoprotein E2 of CSFV. Further researches will be needed to explore more mechanisms of CSFV infection and proliferation.

## Conclusion

Our work shows that integrin β3 is positively correlated to CSFV proliferation, that integrin β3 is up-regulated post CSFV challenge and that depletion of integrin β3 decreases CSFV infection and proliferation. Therefore, integrin β3 is revealed to be required for CSFV infection and proliferation. Thus integrin β3 could be a viable target for antiviral therapies to control CSFV infection.
